# Molecular Characterization of Corynebacterium diphtheriae isolates, Russia, 1957–1987

**DOI:** 10.3201/eid0805.010276

**Published:** 2002-05

**Authors:** Vegard Skogen, Valentina V. Cherkasova, Nina Maksimova, Chung K. Marston, Haakon Sjursen, Michael W. Reeves, Ørjan Olsvik, Tanja Popovic

**Affiliations:** * University of Tromsø, Norway; † G. N. Gabrichevsky Institute for Epidemiology and Microbiology, Moscow, Russia; ‡ Centers for Diseases Control and Prevention, Atlanta, Georgia, USA; § University of Bergen, Haukeland Hospital, Bergen, Norway

**Keywords:** Corynebacterium diphtheriae, molecular epidemiology, Russia

## Abstract

In the 1990s, the Newly Independent and Baltic States of the former Soviet Union experienced the largest diphtheria outbreak since the 1960s; it was caused by Corynebacterium diphtheriae strains of a unique clonal group. To address its origin, we studied 47 clinical isolates from Russia and demonstrated that this clonal group was an integral part of the endemic reservoir that existed in Russia at least 5 years before the epidemic began.

In the pre-vaccine era, diphtheria was a major cause of childhood illness and death worldwide. After the diphtheria toxoid vaccine was introduced, a decline in diphtheria cases was seen where the vaccine was used. In some areas of the Soviet Union, diphtheria vaccination started as early as the 1920s, but it was not included in the general immunization program for children until 1958 (*1*). After 1958, reported diphtheria cases declined steadily except for a small increase in incidence during the 1980s and the epidemic that started in 1990. In 1991, after the breakup of the Soviet Union, routine childhood vaccination programs were disrupted due to interruption of vaccine supplies to countries in Central Asia, the Caucasus, and the Baltic region. A major diphtheria epidemic began in Russia in 1990; during the next 4 years, it reached all the Newly Independent States and Baltic States of the former Soviet Union (FSU) (*1*,*2*). The European Regional Office of the World Health Organization (WHO) now considers this diphtheria outbreak, which resulted in more than 150,000 cases and 4,000 deaths, to be nearly under control (*1*). Several factors, such as an increased proportion of susceptibles in the population, migration, and a deteriorating health infrastructure, are suspected to be major catalysts for this outbreak (*2*). However, the role of biological factors of the causative organism is not clear.

To assess the genetic diversity and structure of the bacteria and its toxin, different molecular typing methods have been used successfully as a complement to traditional characterization (*3*–*6*). Popovic et al. and de Zoysa et al. identified a particular epidemic clonal group, characterized by ribotyping, multilocus enzyme electrophoreses (MEE), and pulsed-field gel electrophoresis (PFGE), associated with the appearance and spread of this outbreak (*7*,*8*). Our study focuses on the origin of this epidemic clonal group and is the first to include a limited number of archival isolates collected more than 30 years before this outbreak began.

## The Study

A convenience sample of 47 Corynebacterium diphtheriae isolates was available for analysis from a collection of isolates obtained during 1957-1987, before the onset of the recent diphtheria outbreak. These isolates were collected from both carriers (n=37) and patients (n=10) in different regions of Russia. All isolates were kept freeze-dried at the G. N. Gabrichevsky Institute for Epidemiology and Microbiology, Moscow, Russia, and were transported on silica gel packages to the Centers for Disease Control and Prevention, Atlanta, Georgia, for molecular characterization.

All isolates were biotyped by using the commercial API Coryne kit (Biomerieux, Lyon, France). Toxigenicity status was determined by the Elek test, as recommended by WHO (*9*), and by the polymerase chain reaction (PCR), which targeted both A and B subunits of the tox gene (*10*).

All the strains were characterized by ribotyping as previously described (*11*). The hybridization was done by using five oligonucleotide probes according to Regnault et al. (*12*). Ribotyping pattern designations were based on the scheme established by Popovic et al. (*7*). A difference in one band was defined as an individual ribotype (RT).

MEE was carried out as previously described (*7*,*11*). The electromorphs of the same enzyme were visualized in a starch gel matrix as bands of different migration rates. Each electromorph was considered to represent a distinct allele of the same enzyme. By testing 27 different enzymes, a profile of electromorphs, defining the electrophoretic type (ET) of each strain, was obtained. The genetic relatedness of ETs was illustrated as a dendrogram, which was generated by the average-linkage method of clustering ETs described by Selander et al. (*13*).

We examined 47 C. diphtheriae isolates collected in the pre-epidemic period (1957-1987) from 10 patients and 37 carriers in different areas of Russia. Thirty-nine strains were of the gravis biotype, 7 were the mitis biotype, and 1 was of the intermedius biotype. All the mitis biotype strains were toxigenic. Among the gravis biotype strains, 36 were toxigenic, and 3 were nontoxigenic. No discrepancies between the results obtained by traditional identification, the API Coryne test, or toxigenicity testing by the Elek test and PCR were detected.

**Figure 1:**
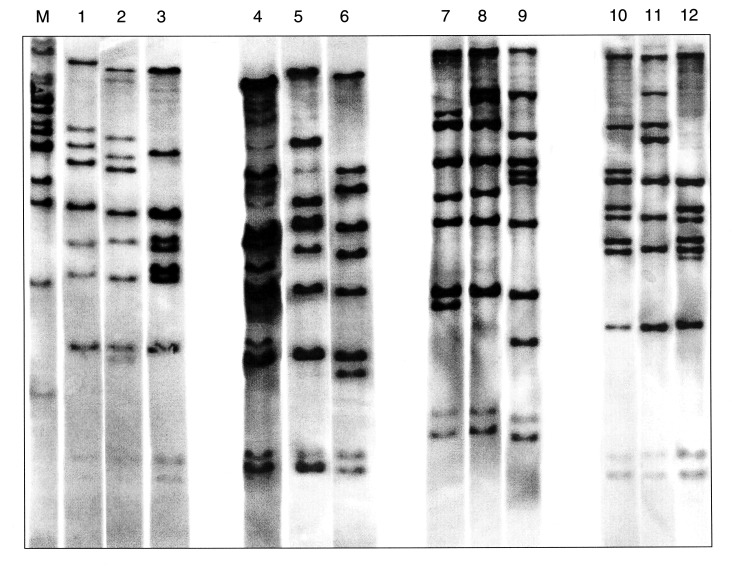
Twelve BstEII ribotypes identified in 47 Corynebacterium diphtheriae isolates collected in the Russian Federation between 1957 and 1987. The figure is composed of ribotype gels exemplifying the different patterns observed in the strain collection. Lane M, molecular weight marker; lane 1, ribotype M11e; lane 2, M11f; lane 3, M13a; lane 4, M7a; lane 5, unique; lane 6, G4; lane 7, unique; lane 8, M11g; lane 9, M3; lane 10, M1b; lane 11, M6; lane 12, M13b.

In the 47 isolates, 12 different RTs were identified (Figure 1). Twenty-two (47%) were of the M11e RT; all were toxigenic and of the gravis biotype. They were collected from 1957 to 1985. RT G4, characteristically seen in the recent epidemic clonal group, was identified in 6 (13%) isolates, all of which were collected from 1984 through 1987. Four isolates had two new ribotype patterns, not previously described. They were collected from 1977 through 1981. RT G4v, a variant of the G4 RT, was identified in a single strain isolated in 1977. Three of the isolates (all toxigenic and of the mitis biotype) had a unique RT not previously described; they were collected from 1977 through 1981.

**Figure 2:**
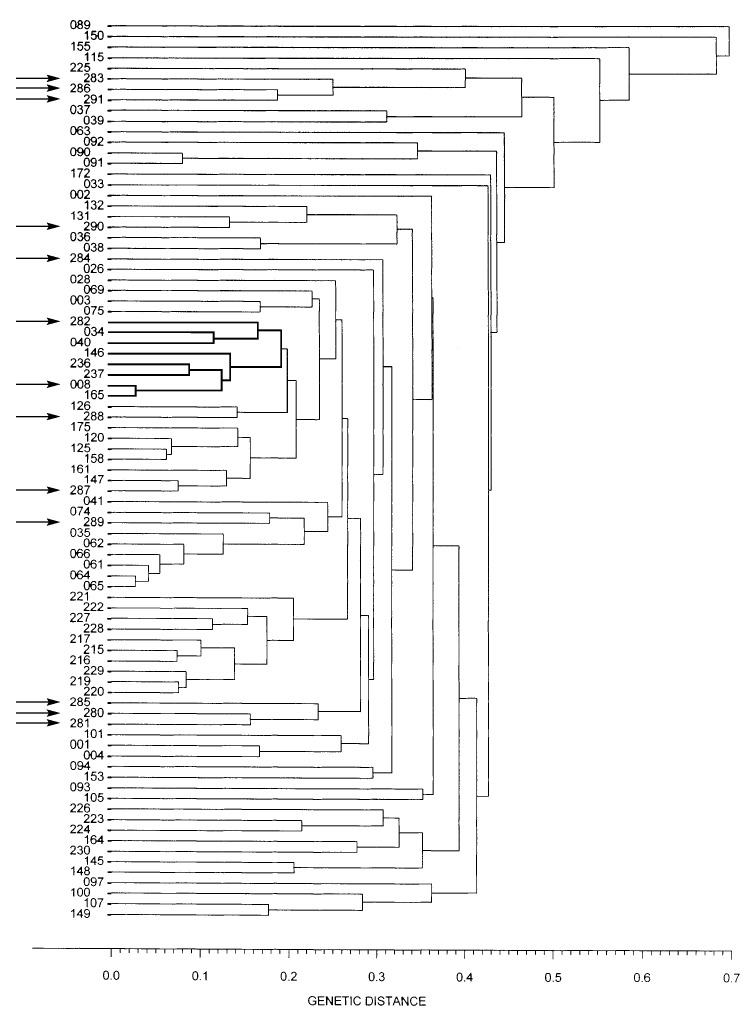
Dendrogram showing the genetic relatedness of 85 electrophoretic types (ETs) of Corynebacterium diphtheriae isolates collected in different countries around the world. Arrows indicate the different ETs identified among the 47 C. diphtheriae isolates. The ET8 complex is marked with thicker lines.

Sixteen (6 isolates of RT pattern G4 and 10 isolates of different RT patterns) of the 47 isolates were analyzed by MEE; 13 different ETs were identified. Of the six isolates with the G4 patterns, four also belonged to the ET8 complex (Figure 2). An additional isolate (strain designation B533 in Table) collected in 1957 belonged to the ET8 complex but had a different RT.

**Table:** Designations and characteristics of 47 Corynebacterium diphtheriae strains collected in Russia, 1957–1987

Ribotype	Geographic area of isolation	No. isolates	Year of isolation	Biotypea	ETb
G4	Anapa	1	1984	G	286
	Moscow	2	1985, 1987	G	291, 8
	Smolensk	1	1985	G	8
	Sverdlovsk	2	1987	G	8
M1b	Anapa	1	1984	M	287
M3	Krasnoyarsk	2	1979	M	290, ND
	Ivanov	1	1976	M	ND
M6	Moscow	1	1981	G	ND
M7a	Moscow	2	1972, 1973	G	ND
M11e	Moscow	13 1	1964-1977 1964	G G	ND 283
	Vladivostok	2 1	1957 1957	G G	280, 281 ND
	Buryatiya	1	1976	G	285
	Groznyi	1	1985	G	ND
	Vladimir	1	1977	G	ND
	Tatarstan	1	1977	G	ND
	Omsk	1	1976	G	ND
M11f	Vladivostok	1	1957	G	ND
	Omsk	2	1977	Ge	ND
M11g	Kirov	1	1978	G	ND
M13a	Vladivostok	1	1957	Ge	282
	Vladimir	1	1976	G	284
	Krasnoyarsk	1	1979	G	289
M13b	Moscow	1	1981	Id	ND
Newc	Vladivostok	1	1981	M	ND
	Moscow	1 1	1977 1977	M Ge	ND 288
	Krasnoyarsk	1	1979	M	ND

a G, biotype gravis; M, biotype mitis; I, biotype intermedius. b ET, electrophoretic type. c New ribotype, pattern has not been previously observed. dND, not done. eNontoxigeic by the Elek test and polymerase chain reaction

## Conclusions

In the pre- and early vaccine era, diphtheria incidence was high in the Soviet Union. After the diphtheria vaccine was introduced, a decrease in incidence was seen in the 1950s. During the mid-1970s, immunization programs resulted in control of diphtheria throughout the country. However, an increase in incidence was noted at the end of 1970 and during the 1980s, and a peak was observed in 1983. This resurgence was associated with a change in the biotype of the circulating C. diphtheriae strains from gravis, which had been dominating for several decades, to mitis (*14*).

To allow better monitoring of the global spread of diphtheria, the WHO ribotyping database for C. diphtheriae was established at the Pasteur Institute in Paris, France. The institute demonstrated that C. diphtheriae RTs are quite diverse worldwide but remain stable over time (*15*). Both ribotyping and MEE have provided a significant level of differentiation and reliability and subsequently have been accepted as the standard for molecular subtyping of C. diphtheriae. Thus, we used these molecular methods to characterize our archival isolates.

Twelve different RTs were found in our 47 isolates. Our data show that nine C. diphtheriae isolates from the 1950s and 1960s had an RT pattern (M11e) that was very similar to ribotype M11, which was only seen occasionally in the FSU in the 1990s. Epidemic RT G4 was seen in six toxigenic C. diphtheriae isolates collected from 1984 through 1987 in four distant regions of Russia (Moscow and Moscow region, Anapa, Smolensk, and Sverdlovsk) from both diphtheria patients and carriers; four of these isolates were also members of the ET8 complex.

Our investigation of the origin of the epidemic clonal group determined that, in our strain collection, the earliest reported strain of this clonal group was identified in Smolensk in 1985, and that strains of this clonal group were simultaneously present in several geographically distant areas in Russia from 1985 through 1987. These findings suggest that the current epidemic clone was an integral part of the endemic reservoir that existed in the FSU at least 5 years before the epidemic began. Further studies that would include a large number of gravis biotype strains from throughout the Soviet Union isolated from 1980 through 1985 might unveil where and when strains of the epidemic clone were first associated with disease or carriage.

## References

[1] Vitek CR, Wharton M. Diphtheria in the Former Soviet Union: re-emergence of a pandemic disease. Emerg Infect Dis. 1998;4:539–50. 9866730 10.3201/eid0404.980404 PMC2640235

[2] Dittman S, Wharton M, Vitek C, Ciotti M, Galazka A, Guichard S, Successful control of epidemic diphtheria in the states of the former union of Soviet Socialist Republics: lessons learned. J Infect Dis. 2000;181:S10–22. 10.1086/315534 10657185

[3] Pappenheimer AM, Murphy JR. Studies on the molecular epidemiology of diphtheria. Lancet. 1983;2:923–6. 10.1016/S0140-6736(83)90449-X 6138500

[4] Bobkova MR, Komborarova SI, Lipis SV, Bobkova AF, Mazurova IK. The use of DNA fingerprint analyses for the differentiation of populations of toxigenic Corynebacterium diphtheriae. Zh Mikrobiol Epidemiol Immunobiol. 1989;7:28–30. 2530743

[5] Rappuoli R, Perugini M, Ratti G. DNA element of Corynebacterium diphtheriae with properties of an insertion sequence and usefulness for epidemiological studies. J Bacteriol. 1987;169:308–12. 3025175 10.1128/jb.169.1.308-312.1987 PMC211769

[6] Reacher M, Ramsay M, White J, De Zoysa A, Efstratiou A, Mann G, Nontoxigenic Corynebacterium diphtheriae: an emerging pathogen in England and Wales. Emerg Infect Dis. 2000;6:640–5. 11076724 10.3201/eid0606.000614 PMC2640921

[7] Popovic T, Kombarova SY, Reeves MW, Nakao H, Mazurova IK, Wharton M, Molecular epidemiology of diphtheria in Russia, 1985-1994. J Infect Dis. 1996;174:1064–72. 8896510 10.1093/infdis/174.5.1064

[8] De Zoysa A, Efstratiou A, George RC, Jahkola M, Vuopio-Varkila J, Deshevoi S, Molecular epidemiology of Corynebacterium diphtheriae from North-western Russia and surrounding countries studied by using ribotyping and pulsed-field gel electrophoresis. J Clin Microbiol. 1995;33:1080–3. 7615709 10.1128/jcm.33.5.1080-1083.1995 PMC228108

[9] Efstratiou A, Maple PA. WHO manual for the laboratory diagnosis of diphtheria. Geneva: World Health Organization, 1994, no. ICP-EPI 038(C).

[10] Mikhailovich V, Melnikov V, Mazurova I, Wachsmuth JD, Wenger M, Wharton M, Application of PCR for detection of toxigenic Corynebacterium diphtheriae strains isolated during the Russian diphtheria epidemic, 1990 through 1994. J Clin Microbiol. 1995;33:3061–3. 8576378 10.1128/jcm.33.11.3061-3063.1995 PMC228639

[11] Popovic T, Kim C, Reiss J, Reeves M, Nakao H, Golaz A. Use of molecular subtyping to document long-term persistence of Corynebacterium diphtheriae in South Dakota. J Clin Microbiol. 1999;37:1092–9. 10074531 10.1128/jcm.37.4.1092-1099.1999 PMC88654

[12] Regnault B, Grimont R, Grimont PAD. Universal ribotyping method using a chemically labelled oligunucleotide probe mixture. Res Microbiol. 1997;148:649–59. 10.1016/S0923-2508(99)80064-3 9765850

[13] Selander R, Caugant DA, Ochman H, Musser JM, Golmour MN, Whittam TS. Methods of multilocus enzyme electrophoresis for bacterial population genetics and systematics. Appl Environ Microbiol. 1986;51:873–84. 2425735 10.1128/aem.51.5.873-884.1986 PMC238981

[14] Markina SA, Maksimova NM, Vitec CR, Bogatyreva EY, Monisov AA. Diphtheria in the Russian Federation in the 1990s. J Infect Dis. 2000;181(Suppl 1):S27–34. 10.1086/315535 10657187

[15] Grimont P, Grimont F, Collin M, Ruckly C, Martin-Delautre S, Regnault B, The Corynebacterium diphtheriae ribotype database project. In: Program and abstracts of the Sixth International Meeting of the European Laboratory Working Group on Diphtheria. European Commission, Brussels, Belgium, 2000.

